# Editorial: Women in microbial symbioses: 2022/2023

**DOI:** 10.3389/fmicb.2024.1393685

**Published:** 2024-03-13

**Authors:** Alejandra Prieto-Davó, Rosario Gil

**Affiliations:** ^1^Chemistry Unit in Sisal, Faculty of Chemistry, National Autonomous University of Mexico, Mexico City, Mexico; ^2^Institute for Integrative Systems Biology (I2SysBio), University of Valencia and Spanish Research Council, Paterna, Spain

**Keywords:** microbial symbioses, women in science, symbionts, microbiomes, bacteria

The International Day of Women and Girls in Science is observed annually on February 11th worldwide. Having a dedicated day underscores the imperative need to rectify the gender imbalance in Science, Technology, Engineering and Mathematics (STEM) disciplines to harness the full spectrum of talent and expertise. Women and girls represent half of the world's population and consequently half of its potential across all fields. While progress has been made in recent years to engage women and girls in STEM careers, with 54% of the total human resources (from 25 to 64 years old) in science and technology within the European Union being women in 2021 [Instituto Nacional de Estadística, INE (Spain), 2022], the situation varies significantly in regions like Latin America, Asia, and Africa where the representation of women researchers averages around 30% (GenderInSITE, 2021). Despite progress, recruiting and retaining women in scientific fields remain challenging. For instance, in Mexico, only 13.5% of professional women pursue STEM-related degrees (García Dobarganes and Masse Torres-Tirado, 2022). Moreover, globally, there's a notable decline over time as women juggle family responsibilities and work, often encountering barriers that impede their advancement toward higher positions in their academic and professional STEM trajectories—a phenomenon termed the “leaky pipeline” (Breaking the Bias in Microbiology, 2022).

Although many pioneering women scientists laid the groundwork for the development of studies on microbial symbiosis throughout the last decades of the past century, before omics techniques emerged as one of the main tools for understanding this fascinating subject, their contributions are often overlooked. This Research Topic aims to address gender inequities by spotlighting the work of women involved in the study of symbiotic relationships with microorganisms.

Symbiosis denotes a stable association between two or more organisms from different species, wherein at least one benefits, regardless of the fitness outcome for the other partner(s). Often, these relationships involve a eukaryote and one or many prokaryotes (Mendes et al., 2013; Raina et al., 2018; Perreau and Moran, 2022). The advent of genomics and high-throughput sequencing has revolutionized the study of these interactions, as evidenced by the diverse array of topics covered in this Research Topic—from phages associated with rhizobia symbionts to microbial interactions with marine invertebrates, livestock, and even humans.

The coral microbiota is an important focus of interest, since bleaching due to the loss of their photosynthetic endosymbionts is one of the consequences of global warming and represents a threat to these colonial marine invertebrates. Lima et al. shed light on the effects of a changing and warming ocean on the *Mussismilia* coral symbiont *Cladocopium* and compared thermally tolerant vs. sensitive strains. They report changes in cell densities, cell wall thickness, lipids, O_2_, proteins and pigments, which suggest a negative effect of thermal stress in *Cladocopium*.

Also related to the marine ecosystems, the microbiota of the moon jellyfish *Aurelia aurita* is studied by Weiland-Bräuer et al. through a transcriptomic approach focusing on the impact of microbiome disturbance on host gene expression. The native microbiome has been manipulated in polyps both by reducing it through an antibiotic treatment and increasing the load of potential pathogens. Special attention is paid to genes involved in immune response and defense strategies, including quorum quenching as a fast response to potential pathogens. The study also highlights the importance of maintaining a balanced microbiome for morphogenesis and development.

The rhizosphere is the soil environment where plant roots interact and establish a complex symbiosis with a rich microbial community. It is represented in this Research Topic by the work of Klonowska et al. who studied an additional level of integration widely found in nature: the presence of viruses in the bacterial genomes and their possible implication in the symbiotic relationship with the host. The work included here explored the genome of a cultivated rhizobial Betaproteobacteria strain from *Mimosa pudica*, and described a Mu-like capsular phage and one filamentous phage for the first time. Further investigation was unable to detect any filamentous prophages in rhizobial alphaproteobacteria, while they were commonly found in other rhizobial betaproteobacteria.

The composition of the vaginal microbiota plays a key role in females' health and varies during their reproductive life. Zhang et al. explore the potential association between the impairment of pelvic muscle functions postpartum and the changes in the vaginal microbiota composition. They observed that women with compromised pelvic muscle functions showed a decrease in the abundance of *Lactobacillus* and a significant increase in species richness and diversity, so that the restoration of pelvic functions may have a positive impact in recovering a balanced vaginal microbiota.

While the human vaginal microbiota is being widely studied, Barba et al. present a pioneering work that gives a first glimpse into the vaginal microbiota dynamics of livestock mammals during the reproductive cycle. Using 16S rRNA gene amplicon sequencing, their study of the microbial community profiling in nulliparous ewes and the foreskin of rams suggest that variations in the abundance of certain phyla, such as Firmicutes, could be related to infertility, that pregnant ewes regulate microbial abundances to have a more balanced state, and that ram's preputial microbiota can influence the vaginal microbiota through natural mating.

These five articles provide a glimpse into the vast research in symbiosis that women are either heading or participating in. It's a microbial world and the relationship of the eukaryotic organisms with their microbiota is beginning to shed information on responses to environmental pressures, and many women have contributed to the fundamental understanding of these relationships. Furthermore, the more we know about the microbes that live within us, the better we will understand our health and responses to medical treatments, an area that crucially needs to be addressed with a gender perspective.

## Author contributions

AP-D: Supervision, Writing—original draft, Writing—review & editing. RG: Writing—original draft, Writing—review & editing.
